# Adherence to Exercise Training Within a Multimodal Prehabilitation Program: An Exploratory Study of Influencing Factors

**DOI:** 10.3390/jcm14113813

**Published:** 2025-05-29

**Authors:** Raquel Risco, Raquel Sebio-García, Rubèn González-Colom, Marta Ubré, Fernando Dana, Edgar Iglesias-García, Graciela Martínez-Pallí, on behalf of the Hospital Clínic de Barcelona Prehabilitation Group

**Affiliations:** 1 Anesthesiology Department, Hospital Clínic de Barcelona, 08036 Barcelona, Spain; rrisco@clinic.cat (R.R.); mubre@clinic.cat (M.U.); fjdana@clinic.cat (F.D.); 2 August Pi i Sunyer Biomedical Research Institute (IDIBAPS), University of Barcelona, 08036 Barcelona, Spain; sebio@clinic.cat (R.S.-G.); rgonzalezc@recerca.clinic.cat (R.G.-C.); 3 Physical Medicine and Rehabilitation Department, Hospital Clínic de Barcelona, 08036 Barcelona, Spain; ediglesias@clinic.cat; 4 Biomedical Research Networking Center on Respiratory Diseases (CIBERES), 28029 Madrid, Spain

**Keywords:** prehabilitation, patient adherence, exercise program

## Abstract

**Background/Objectives:** The real impact of prehabilitation in the healthcare setting is controversial due to the efficacy–effectiveness gap. The effectiveness of prehabilitation in real-world scenarios has been associated with program attrition and adherence. This study aimed to identify factors influencing adherence to a multimodal prehabilitation program for patients undergoing major surgery. **Methods:** This is a analysis of a prospective trial conceived to explore the implementation of prehabilitation in a real-life setting. Participants were patients enrolled in our multimodal prehabilitation program, candidates for major surgery, and at high risk for postoperative complications. Sociodemographic and clinical variables were studied, with adherence to the program as the primary outcome. Descriptive analyses were conducted to examine associations between adherence and the study variables. A binary logistic regression model was applied to identify predictors of adherence. **Results:** Among the 559 patients included in the study, 356 (63.7%) were labelled as adherent. The analysis revealed significant associations between adherence and working status, type of exercise program prescribed (*p* < 0.001), smoking status (*p* = 0.023), age (t = −3.00, *p* = 0.003), comorbidities (t = −2.19, *p* = 0.029), and self-reported physical activity (t = −2.45, *p* = 0.015). The logistic regression identified as independent factors the type of exercise prescription, smoking status, residential area, working status, and neoadjuvant therapy. The predictive model demonstrated good specificity (86.1%) but lower sensitivity (50.6%), suggesting its utility in identifying patients at risk of non-adherence. **Conclusions:** Multiple factors influence adherence in prehabilitation programs. Our model exhibited good accuracy and specificity, but poor sensitivity.

## 1. Introduction

Multimodal prehabilitation has emerged in recent years as an innovative approach to enhance patients’ physiological and psychological resilience before surgery. This patient-tailored, holistic, preventive, and short-term program aims to improve aerobic capacity, nutritional balance, and psychological well-being. Several randomized controlled trials and meta-analyses have demonstrated its effectiveness in reducing postoperative complications and improving functional recovery [1,2,3,4]. However, its real impact in the healthcare setting is controversial due to the well-known efficacy–effectiveness gap observed between what can be found in a controlled setting such as a clinical trial and what we observe in clinical practice [5]. This gap may be even more pronounced for complex interventions like exercise, which require active patient participation, and could hinder the widespread adoption of programs such as prehabilitation, thereby diminishing their positive outcomes.

A recent study [6] conducted by our group identified two main factors influencing prehabilitation effectiveness in real-world scenarios, namely (i) program attrition and adherence; and (ii) degree of surgical complexity, with the intervention being more effective in reducing morbidity and decreasing costs among those completer patients undergoing highly complex surgeries. While the surgical insult is a unmodifiable variable, adherence and completion with the prehabilitation program can be optimized using several tactics.

There is no consensus as what constitutes compliance or good adherence within a prehabilitation program, but various trials define an adherent participant as one who completes at least 80% of the prescribed sessions or tasks [7,8]. In our last publication [6], we considered a patient as a “prehabilitation completer” if the following criteria were met: (i) a minimum program duration of four weeks; and (ii) high adherence (≥80%) to the exercise prescribed. Unfortunately, according to our results, only 34% of patients met both conditions. When analyzed separately, we observed that program duration was most commonly affected by factors outside the patients’ control, such as changes in the surgical program or disease progression, which prevented us from adopting any potential measures of improvement. However, adherence to a behavioral intervention such as prehabilitation exercise has more potential to be modulated as it is likely associated with a wider range of factors [9,10]. Logistical barriers, lack of motivation, and physical or cognitive limitations have been identified as factors affecting adherence. Recognizing and understanding these factors allows designing interventions that enhance patient participation and, consequently, clinical outcomes [11]. In this context, the conceptual framework of the “Learning Rehabilitation System” proposed by Bickenbach et al. highlights the importance of person-centered approaches tailored to the patient’s environment. This model emphasizes the need to consider contextual, ethical, and systemic factors in the planning and implementation of rehabilitation programs, including prehabilitation [12]. The integration of this approach may be key to addressing the geographical and social disparities that affect the adherence and effectiveness of these programs [13].

Therefore, the aim of this study was to identify potential factors which could be associated with patient adherence to the exercise component of a multimodal prehabilitation program. We hypothesized that several clinical and sociodemographic factors could influence the adherence rates and thus could be targeted in future interventions to increase response to the intervention.

## 2. Materials and Methods

### 2.1. Study Design

This is an analysis stemming from a large, prospective study conducted as part of the implementation process within our prehabilitation unit in a real-life setting at Hospital Clínic Barcelona (HCB). The analysis covering the preceding period, spanning from June 2017 to December 2019, has been published previously [6]. The Ethics Committee for Medical Research of HCB approved the study (HCB/2019/1030; 28 September 2021). An informed consent was understood, accepted, and signed by all subjects included in the trial. The study was conducted in accordance with the principles set forth in the Helsinki Declaration.

### 2.2. Participants

Participants involved in this study were consecutive patients referred and enrolled in our multimodal prehabilitation between December 2020 to June 2024. The attending surgeon referred patients to the program based on the following pre-established criteria: (i) being a surgical candidate for major digestive, cardiothoracic, gynecological, or urological surgery; (ii) being at high risk for postoperative complications defined by age ≥ 70 and/or American Society of Anesthesiologists (ASA) risk scale 3 to 4 [14], and/or patients suffering from severe deconditioning caused by cancer and/or undergoing highly aggressive surgeries (grade III according to the modified Johns Hopkins criteria for surgical invasiveness) [15]; and (iii) having an expected preoperative schedule allowing for at least 4 weeks of prehabilitation without unnecessary delays to the surgical program. Exclusion criteria were (i) non-elective surgery; and (ii) no possibility or willingness to attend the appointments (particularly supervised exercise training).

### 2.3. Intervention

The multimodal prehabilitation program provided, in addition to the standard of care (medical management of multimorbidity and promotion of a healthy lifestyle), the following interventions: (i) motivational interviewing to promote patient engagement with the program and patient empowerment; (ii) a goal-setting physical activity program at home (PA-based program); (iii) hospital-based supervised exercise training two to three times per week (ET-based program); (iv) nutritional or dietary recommendations and/or supplementation; and (v) psychological support (if recommended). Patients who were not able to attend the supervised exercise training at the hospital gym or had a low-risk for cardiovascular events during exercise were only offered the PA-based program. In this group of patients, weekly goals were monitored through a mobile app (PREHAB^®^ https://theprehabguys.com/, accessed on 20 May 2025) connected to a fitness tracker or their phone. Patients who did not have smartphones were given a paper-based diary with an analog pedometer. Patients were asked to track the number of steps taken per week, as well as register any other form of physical activity performed (e.g., stair climbing, stationary bike). In addition, they were also prescribed one day a week of supervised exercise at the hospital gym to monitor progress.

The entire program was conducted by a multidisciplinary team including anesthesiologists, physiotherapists, dietitians, psychologists, and nurses. A more comprehensive description of the program can be found elsewhere [6].

### 2.4. Study Variables

Patients were assessed individually by the multidisciplinary team at the Prehabilitation Unit. Specifically, each patient was individually assessed by an anesthesiologist, physiotherapist, nutritionist, psychologist, and nurse, according to their respective area of expertise. Baseline measurements encompassed the following elements: (i) comorbidities (Charlson Comorbidity Index) [16]; self-reported functional capacity (Duke Activity Status Index [DASI]) [17] and frailty (Clinical Frailty Scale [CFS]) [18]; (ii) nutritional status based on the Global Leadership Initiative on Malnutrition (GLIM) criteria [19], albumin and hemoglobin levels, and body mass index; anxiety and depression according to the Hospital Anxiety and Depression Scale (HADS) [20]; and a battery of physical tests, including a handgrip strength test, the 6-Minute Walk Test (6MWT) [21], the 30 s Sit-to-Stand test (30″ STS) [22], and self-reported physical activity levels using the Yale Physical Activity Survey (YPAS) [23].

In addition, we collected a series of sociodemographic and clinical variables to characterize the sample, including (i) age, (ii) gender; (iii) highest level of education; (iv) area of residency; (v) living status; (vi) civil status; (vii) working status; (viii) means of transportation; (ix) type of surgery; (x) surgical risk (ASA score); (xi) neoadjuvant therapy; (xii) polypharmacy; and (xiii) smoking status.

### 2.5. Outcomes

Adherence to the exercise component of the prehabilitation program was the main outcome in this study. For patients in the ET-based program, adherence was calculated from the number of sessions attended in relation to the number of prescribed sessions. For patients in the PA-based program, adherence was calculated considering the number of steps taken in a given week in relation to the goal set, as well as attendance of the weekly supervised exercise session. International recommendations on step-based physical activity [24] were used as a theoretical framework to set up the objective, and the step goal was to increase daily steps by 500 to 1000 compared to the previous week. Consistent with previous studies [6,7,8], we considered a patient as adherent if attendance reached ≥80% of the prescribed training sessions and/or ≥80% of the weekly step count goal was met.

### 2.6. Data Analysis

Descriptive measurements were obtained for the main variables in the study. Normality was checked using the Kolmogorov–Smirnov test for each independent variable of interest. Mean (SD) or median (Q1-Q3) were calculated based on distribution. Based on our prior experience [6], we classified the patients into two categories, which were not mutually exclusive: (i) prehabilitation-completer, as in a patient that engaged in at least 4 weeks of prehabilitation and; (ii) prehabilitation-adherent, as in a patient who achieved ≥80% of the prescribed training sessions or physical activity goals. As program completion is highly non-volitional (e.g., changes in surgical schedule or treatment plan), we therefore focused on analyzing only the factors associated with adherence ≥80%. The classification into two adherence categories was based both on our prior experience and on the need for a pragmatic binary outcome to guide clinical decision-making and prediction modeling.

Initial analyses were conducted to examine 1-to-1 associations between adherence and a wide range of covariates, including type of surgery, ASA classification, age, sex, smoking status, nutritional status, comorbidities, level of education, living status, frailty, physical functioning (DASI score), hemoglobin levels, type of exercise training prescribed (supervised vs. home-based), and physical performance (6MWT and 30″ STS). For categorical variables, distributions in relation to adherence were analyzed using contingency tables and Fisher’s exact test with simulation for large datasets. For numerical variables, parametric (t-tests) or non-parametric (Mann–Whitney U tests) analyses were applied as appropriate, depending on whether the data met normality assumptions, to compare adherent and non-adherent groups.

Subsequently, a binary logistic regression model was applied to identify predictors of adherence ≥80%. Categorical variables were converted into dummy variables to allow their inclusion in the regression model. Numerical variables were standardized through scaling to facilitate comparability across features with different units. To address multicollinearity, linearly dependent combinations of variables were identified and removed from the dataset. A stepwise forward selection approach was used to develop the logistic regression model. Starting with a baseline model without predictors, variables were added one by one based on the Akaike Information Criterion (AIC) to improve the model fit, while avoiding overfitting. Model validation was performed using confusion matrices, and the discriminative capacity was evaluated by calculating the area under the ROC curve (AUC). Data processing and analysis were conducted using SPSS v25 for Windows^®^ (Microsoft©, Redmond, Washington, DC, USA) and R version 4.1.1.

Missing data were handled by excluding cases with unrecoverable missing values in the adherence records and removing any variables with more than 20% missing values. For the one-to-one adherence and covariate analyses, missing values were omitted to avoid distorting the relationships in the data. In the predictive modeling, due to the large sample size and the low proportion of registries with missing data (less than 5%) in key predictors, as well as the absence of bias in the missingness patterns, individuals with critical missing predictor values were excluded to ensure data completeness.

## 3. Results

Between December 2020 and June 2024, a total of 782 patients were referred to our prehabilitation program. Of those, data on adherence and compliance were available for 559 (71.5%) patients who were included in the study. Reasons for missing data included patient refusal to engage in the program (n = 11), withdrawal from the program (n = 21), disease progression (n = 23), change in surgical schedule (n = 32), unknown reasons (n = 64), or ongoing program (n = 72). The main characteristics of the patients included can be found in Table 1.

According to our classification criteria, a total of 368 patients (65.7%) completed a minimum of 4 weeks of pre-habilitation, whereas 356 (63.7%) were labelled as adherent (attendance to exercise/physical activity program ≥ 80%). The number of patients who completed 4 weeks of prehabilitation with good adherence was 222 (39.7%).

The analysis of categorical variables revealed significant associations between adherence and three factors: working status (*p* = 0.011), smoking status (*p* = 0.023), and the type of program assigned (*p* < 0.001). Patients in the supervised exercise training group were more likely to achieve adherence levels of ≥80%, as were retired patients. On the other hand, smoking was associated with lower adherence. In addition, there was a strong tendency for gender to be associated with adherence (*p* = 0.052), with more male patients achieving ≥80% adherence compared to women (63.9% vs. 56.7%). For numerical variables, significant differences were found for age (t = −3.00, *p* = 0.003), Charlson Comorbidity Index (t = −2.19, *p* = 0.029), and YPAS scores (t = −2.45, *p* = 0.015) between adherent and non-adherent groups. Specifically, adherent patients were older, had slightly higher comorbidity scores, but reported higher physical activity levels at baseline (Table 2).

**Figure 1:**
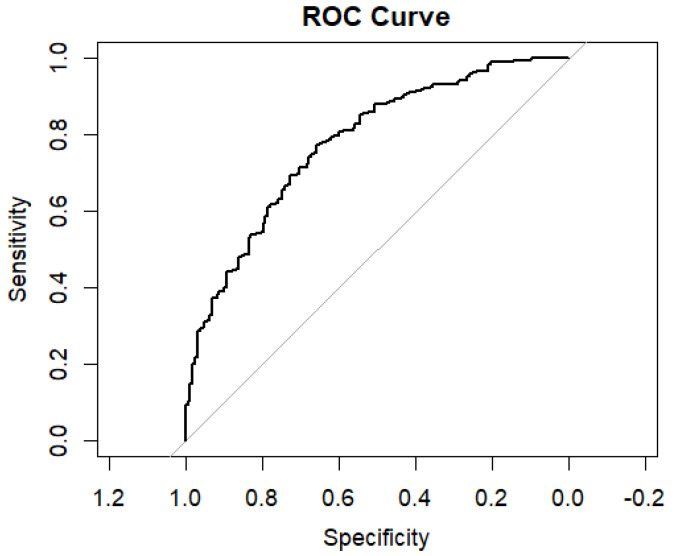
Receiver Operating Characteristic (ROC) curve for the logistic regression model predicting adherence (attendance rate greater than or equal to the 80% of the sessions) to the prehabilitation program.

The logistic regression identified several independent factors associated with adherence. These included the type of exercise prescription (supervised vs. home-based), smoking status, residency area, working status, and neoadjuvant therapy. The model demonstrated good discriminative ability, with an AUC of 0.777 (95% CI: 0.729–0.824). Specificity was high at 86.1%, indicating the model’s strong ability to correctly identify non-adherent patients. However, sensitivity was lower at 50.6%, reflecting moderate performance in detecting adherent patients. The model’s negative predictive value (NPV) was 74.7%, indicating that approximately three out of four times, the model could accurately predict that a patient would not achieve good adherence (Figure 1).

## 4. Discussion

This study explored factors influencing adherence and compliance during prehabilitation in patients scheduled for major surgery, as these were two limiting factors for prehabilitation effectiveness in a previous cohort study [6]. According to our results, several aspects can contribute to decreased adherence to a prehabilitation program, including sociodemographic (working status, age, residential area), clinical (comorbidities, smoking status, neoadjuvant therapy), and procedural (type of program prescribed), which provides valuable information for clinicians involved in delivering these programs to maximize compliance.

Limited literature exists on adherence barriers and facilitators, as well as predicted factors of adherence, in prehabilitation [10]. Most data come from studies conducted in patients with chronic diseases such as Chronic Obstructive Pulmonary Disease (COPD), chronic cardiac disease, or cancer patients undergoing systemic treatment. For instance, in people with COPD, self-efficacy and awareness of the benefits of physical activity have been identified as modifiable factors influencing adherence [25], while other studies have highlighted the role of social support, symptom burden, and program accessibility [26,27,28]. Similarly, in patients with coronary heart disease and depressive symptoms, emotional and motivational barriers have been shown to affect participation in exercise-based interventions [29]. In the oncology setting, recent qualitative evidence has underscored the importance of tailoring exercise during systemic treatment to patient preferences and limitations [30,31], and pilot trials such as the BENITA study have demonstrated the feasibility of combined exercise and nutrition interventions during chemotherapy [32]. In support of these findings, a systematic review and meta-analysis by Bullard et al. [33] reported a mean adherence rate of 77% across physical activity interventions in patients with cancer, cardiovascular disease, and diabetes, reinforcing the relevance of structured and patient-centered approaches to optimizing engagement. Our findings align with previous studies that identified significant predictors of adherence to prehabilitation programs as age and previous level of physical activity [34,35]. Interestingly, older patients were found to be more adherent compared to their younger counterparts. Although this might seem somewhat counterintuitive, as older patients usually suffer from more comorbidities and a higher symptom burden, it could be related to their greater availability to participate in the prehabilitation program. Indeed, this finding aligns with the fact that only 61% of non-adherent patients were retired compared to 74% within the adherent group. As per physical activity, we showed that higher baseline levels of self-reported physical activity were associated with greater odds of having good adherence. We hypothesize that patients who engage in less PA or have no history of previous exercise behavior will be less like to adhere to the program because they might exhibit more perceived barriers to participate in exercise programs [36] or might have less self-efficacy compared to those who are more physically active.

Besides age and PA level (reported through YPAS), the logistic regression identified other independent factors influencing adherence, including type of exercise prescription, smoking habit, area of residence, employment status, and neoadjuvant treatment. Patients in the supervised training group adhered better than those training at home, emphasizing the importance of professional support and structure in the implementation of training programs [37]. Supervision can act as a constant reminder and ensure correct execution of exercises, reducing perceived barriers and increasing participants’ confidence. However, this approach might not work for everyone and could be less feasible in other settings and healthcare systems. For instance, in a study conducted among patients undergoing prehabilitation in an Australian population, Waterland et al. found that patients usually preferred exercise at home compared to face-to-face [38]. This could be related to the large geographical dispersion of patients in some countries such as Australia and the difficulties in commuting to a facility-based exercise program. This was also identified by the inclusion of area of residence as a factor influencing adherence in our study and highlights the need to consider geographic disparities in the design of future interventions, to limit inequalities in the access to prehabilitation.

Finally, our model also identified that current smokers and patients undergoing neoadjuvant treatment were less likely to achieve good adherence. Smoking status can limit participation in physical activity programs, because it can affect exercise capacity by impairing lung function and increasing symptoms of breathlessness [39], in addition it is associated with less healthy lifestyles and increased sedentary lifestyles [40]. Similarly, for patients undergoing neoadjuvant therapy, the symptom burden caused by cytotoxic drugs can impact their mood and energy, and increase fatigue, impairing participation in the program [41]. Nevertheless, some qualitative studies have shown that patients receiving systematic treatment prior to surgery can also perceive the program as a way of coping with the disease [42] and the side-effects of treatment, as well as a positive distraction [43] from the oncologic process. In fact, recent studies suggest that prehabilitation during neoadjuvant therapy can lead to an increase in the pathological response to treatment [44], thus specific actions aimed at improving adherence to these programs in this particular population are of great relevance.

Although the predictive model demonstrated acceptable discriminative ability (AUC = 0.729) and high specificity (86.1%), the sensitivity was relatively low (50.6%). This implies that the model was better at identifying those likely to be non-adherent than adherent (74.7% negative predictive value), reinforcing its usefulness in clinical settings to identify and support patients at higher risk of dropping out of the program.

This study highlights key factors associated with adherence to exercise in prehabilitation, including age, previous physical activity level, smoking habit, employment, neoadjuvant therapy, and exercise modality. While many of these factors are unmodifiable, they can assist in identifying patients who require enhanced support to participate in the program. Implementing diverse strategies like cognitive behavioral therapy, digital monitoring tools, personalized exercise prescriptions, or hybrid delivery models could mitigate logistical and motivational barriers, thereby improving adherence.

### Limitations

This study has some limitations. First, only consenting patients referred by surgeons or anesthesiologists were included. Patients who did not consent or reported logistical challenges to adhering to the program from the outset were not included in the study. This self-selection of participants may affect the generalizability of the results, as it does not address the factors that influenced the acceptance of this therapeutic intervention. Moreover, of the total sample (n = 782), adherence data were only available for 71.5% of patients. The 28.5% missing data was attributable to factors like altered surgical timelines or patient dropout before the four-week mark. This loss of data presents a potential bias, as patients lacking adherence data might have exhibited lower adherence levels.

Second, we opted to define adherence as a dichotomous variable (meeting ≥80% of session attendance and/or weekly targets) instead of a continuous one. Although a linear definition might have allowed for the evaluation of a dose–response relationship, a dichotomous approach was chosen for its clinical significance and consistency with previous research, facilitating comparisons.

Third, adherence to home-based programs is challenging to determine. The reliance on self-reports, the difficulty in objectively verifying exercise performance, and the potential variability in patients’ adherence to instructions could have impacted the accuracy and reliability of the adherence data.

Finally, we considered adherence to the prehabilitation program only in terms of exercise, without taking into account other key components such as nutrition or psychological support. Since prehabilitation is a comprehensive approach that includes several areas of intervention, focusing exclusively on exercise adherence might not fully reflect the overall effectiveness of the program. This limits our ability to assess the full impact of all the strategies used.

Conversely, the study’s strength lies in its reflection of a real-world scenario within the context of a therapeutic intervention where patient volition is crucial.

## 5. Conclusions

The present study aimed to emphasize the importance of considering multiple dimensions, from individual factors to program characteristics and context, to optimize adherence. From a clinical perspective, recognizing non-modifiable predictors of poor adherence can help identify patients who may benefit from enhanced support strategies. Our predictive model may assist clinicians in effectively targeting interventions. Future research should explore strategies to address the identified barriers.

In summary, this study highlights key predictors of adherence to prehabilitation and supports the need for targeted strategies to improve patient engagement and optimize outcomes in real-world settings.

## Figures and Tables

**Table 1:** Characteristics of patients included in the study (n = 559).

Variable	Value ^1^
Sociodemographics	
Age	69.8 (11.4)
Gender	
Male	342 (61.1%)
Female	215 (38.4%)
Living area	
AISBE ^2^	312 (55.7%)
Barcelona	71 (12.7%)
Metropolitan area	63 (11.3%)
Rest of Catalonia	98 (17.5%)
Living status	
Living alone	146 (26.1%)
Co-living	398 (71.1%)
Missing	16 (2.9%)
Level of studies	
No studies	12 (2.3%)
Primary incomplete	45 (8%)
Primary complete	172 (30.7%)
Secondary	194 (34.6%)
University degree	98 (17.5%)
Postgraduate/Doctorate	20 (3.6%)
Missing	19 (3.4%)
Civil status	
Single	53 (9.5%)
Separated/divorced	33 (5.9%)
Widowed	102 (18.2%)
Married/in couple	349 (62.3%)
Missing	23 (4.1%)
Working status	
Actively working	53 (9.5%)
Household labor	23 (5.9%)
Retired	382 (68.2%)
Unemployed	15 (2.7%)
Sick leave	72 (12.9%)
Student/others	5 (0.9%)
Missing	10 (1.8%)
Means of transportation to hospital	
Walking	82 (14.6%)
Public transportation	262 (46.8%)
Own vehicle	186 (33.2%)
Ambulance	17 (3%)
Missing	13 (2.3%)
Needs accompaniment for hospital visit	
Yes	146 (26.1%)
No	398 (71.1%)
Missing	16 (2.9%)
Clinical features	
Type of surgery	
Colorectal surgery	137 (24.5%)
Valve replacement surgery	65 (11.6%)
Esophagectomy	62 (11.1%)
Lung resection surgery	52 (9.3%)
Cystectomy	47 (8.4%)
Gastrectomy	37 (6.6%)
CRS + HIPEC ^3^	29 (5.2%)
CAGB ^4^	21 (3.8%)
Pancreatic Surgery	19 (3.4%)
Other cardiac surgeries	18 (3.2%)
Liver transplantation	13 (2.3%)
Other digestive surgeries	12 (2.1%)
Bariatric surgery	12 (2.1%)
CAGB + valve replacement	8 (1.4%)
Hepatic resection	7 (1.3%)
Heart transplant	7 (1.3%)
Other surgeries	11 (2%)
ASA	
II	162 (28.9%)
III	335 (59.8%)
IV	62 (11.1%)
Frailty (CSF)	
1	16 (2.9%)
2	69 (12.3%)
3	185 (33%)
4	223 (39.8%)
5	14 (2.5%)
6	46 (8.2%)
7	3 (0.5%)
Oncological surgery	
Yes	379 (67.7%)
No	165 (31.4%)
Missing	10 (1.8%)
Neoadjuvant therapy	
Radiotherapy	4 (0.7%)
Chemotherapy	73 (13%)
Chemoradiotherapy	37 (6.6%)
None	431 (77%)
Missing	14 (2.5%)
Polypharmacy (>5 drugs)	
Yes	296 (52.9%)
No	237 (42.3%)
Missing	27 (4.8%)
Body Mass Index (kg/m^2^)	27.9 (7.5)
Hemoglobin (mg/dL)	12.9 (2)
Albumin (mg/dL)	42.4 (4.6)
Charlson Comorbidity Index	5.7 (2.4)
Smoking	
Former smoker	291 (52%)
Current smoker	62 (11.1%)
Non-smoker	195 (34.8%)
Missing	12 (2.1%)
Nutritional Status ^5^	
No malnutrition	373 (66.6%)
Moderate malnutrition	127 (22.7%)
Severe malnutrition	59 (10.5%)
DASI (points)	27.6 (13.6)
6MWT	
≤400 m	167 (29.8%)
>400 m	285 (50.9%)
Missing	108 (19.3%)
Handgrip strength (kg)	
Women	20.2 (5.8)
Men	33.5 (9.1)
Sit-to-Stand (reps)	
Women	10.6 (4.4)
Men	11.4 (4.8)
Physical Activity Levels (YPAS)	
Not physically active (≤38 points)	368 (65.7%)
Physically active (>38 points)	185 (33%)
Missing	7 (1.3%)
Mood (HADS)	
Anxiety	5 (3.7)
Depression	4.1 (3.3)
Total	9.1 (6.3)

^1^ Data presented as mean (SD), unless otherwise indicated. ^2^ AISBE indicates Integral Health Area Barcelona Left. ^3^ CRS + HIPEC indicates Cytoreductive Surgery + Hyperthermic Intraperitoneal Chemotherapy. ^4^ CAGB indicates Coronary Artery Bypass Grafting. ^5^ Based on GLIM criteria.

**Table 2:** Association between adherence and sociodemographic and clinical variables.

Variable	Adherent *	Non-Adherent *	*p* Value
Age	71 (10.8)	67.9 (12.2)	0.002
Gender (% male)	227 (63.9%)	114 (56.7%)	0.056
Living Area			0.552
AISBE ^1^	198 (57.6%)	113 (56.8%)	
Barcelona	46 (13.4%)	25 (12.6%)	
Metropolitan area	35 (10.2%)	28 (14.1%)	
Rest of Catalonia	65 (18.9%)	33 (16.6%)	
Working Status			0.014
Actively working	26 (7.5%)	27 (13.4%)	
Household work	11 (3.2%)	12 (6%)	
Retired	258 (74.1%)	124 (61.7%)	
Unemployed	8 (2.3%)	7 (3.5%)	
Sick leave	44 (12.6%)	27 (13.4%)	
Student/other	0 (0%)	3 (1.5%)	
Neoadjuvant therapy			0.079
Yes	66 (19.1%)	49 (24.6%)	
No	280 (80.9%)	150 (75.4%)	
Charlson Comorbidity Score	5.9 (2.3)	5.5 (2.5)	0.025
Type of exercise program			0.001
Home-based	51 (14.3%)	47 (23.3%)	
Supervised	286 (80.3%)	134 (66.3%)	
Smoking			0.023
Non-smoker	118 (34%)	77 (38.5%)	
Former smoker	198 (57.1%)	93 (46.5%)	
Current smoker	31 (8.9%)	30 (15%)	
YPAS	35.7 (18.3)	32.1 (16)	0.015

* Data presented as mean (SD), unless otherwise indicated. ^1^ AISBE indicates Integral Health Area Barcelona Left.

## Data Availability

The data associated with the paper are not publicly available but are available from the corresponding author on reasonable request.
